# Supplementary material for: Factors associated with unmet need for family planning in sub-Saharan Africa: A multilevel multinomial logistic regression analysis
Source: PLoS One. 2022 Feb 10;17(2):e0263885. doi: 10.1371/journal.pone.0263885 (PMC8830726; doi:10.1371/journal.pone.0263885)
Supplement: Attachment — Submitted filename: unmet need for FP comments.docx [file pone.0263885.s001.docx]

***General comment***

The author is expected to state whether or not there is used/followed guideline such as Strengthening the Reporting of Observational Studies in Epidemiology (STROBE) or others to conduct this study and write the manuscript. Using such guidelines will enhance the validity of the report.

***Specific comments***

Comment 1

Abstract (background)- the author wrote the facts (the figure) regarding the unmet need for FP. However, the author did not state the research gap. Therefore, reconsider it and add a sentence that indicates the research gap.

Comment 2

Abstract (conclusion)- the conclusion should be in line with the objective of the study. In this case, the author has two major objectives as a research question (i.e., magnitude and factors associated with unmet need). Therefore, the author has to conclude to those major research questions.

Comment 3

Explanatory variables-the author has indicated that both individual and community-level variables were identified after searching different kinds of literature. However, since the source of data is DHS, incorporating explanatory variables from other sources is impossible as we have to only rely on the variables that can be obtained from this secondary data. Therefore, re-write the sentence as: *“Both individual and community level variables were incorporated as explanatory variables after reviewing the DHS data”.*

Comment 4

Data management and statistical analysis- the author used so many variables for the multivariable analysis. As a result, the chance of having multicollinear variables will be increase. Therefore, the author has to report information regarding multicollinearity and the used technique (s) to diagnose it.

Comment 5

Ethical consideration- specify the date when the data were requested and accessed from the DHS program.

Comment 6

Discussion- reduce the first paragraph only to the objective of the study. Other points written here seem results. Hence, move and reshuffle it under the result section.

Comment 7

Discussion- the author has put justification for every comparable or contrasting result. This is very appreciable. However, the justification sentences have to be short and precise. Therefore, chop those sentences into short form. Also, put references for some of your justifications.

Comment 8

Conclusion- the author put the findings (i.e., the prevalence of unmet need for FP and its correlates) of the study as a conclusion. However, the conclusion needs to have a public health implication which is not stated in this manuscript. Public health implication means what does the prevalence indicate according to the current standards of service (national/global target, plan, etc.)? Again, does your finding is low or high according to the current standards of service? Therefore, the author has to forward a valid conclusion based on the actual findings.
